# The Density of Different Local Anesthetic Solutions, Opioid Adjuvants and Their Clinically Used Combinations: An Experimental Study

**DOI:** 10.3390/ph14080801

**Published:** 2021-08-16

**Authors:** Tomasz Jasinski, Dorian Migon, Krystian Sporysz, Wojciech Kamysz, Radoslaw Owczuk

**Affiliations:** 1 Department of Anesthesiology and Intensive Therapy, Medical University of Gdansk, 80-214 Gdansk, Poland; tjasinski@uck.gda.pl (T.J.); krystian.sporysz@gumed.edu.pl (K.S.); 2 Department of Inorganic Chemistry, Medical University of Gdansk, 80-416 Gdansk, Poland; dorian.migon@PolpharmaBiologics.com (D.M.); wojciech.kamysz@gumed.edu.pl (W.K.)

**Keywords:** spinal anesthesia, opioid adjuvants, block distribution

## Abstract

Various opioids are added to local anesthetic solutions for spinal anesthesia. This may change the final density of the local anestetic (LA) mixture. This effect regarding current concepts in spinal anesthesia needs to be re-evaluated. In order to re-evaluate such effects, hyperbaric and isobaric local anesthetic (LA) solutions were mixed with opioid adjuvants (A) using the equipment available in the operating room. Ten density measurements for each composition (LA-A) were performed. The density change of 0.0006 g/mL was regarded as significant. Measured densities were also compared with theoretical values calculated using Hare’s. As a result, the addition of an opioid adjuvant caused a significant reduction in the final density of the LA-A solution. In hyperbaric LA mixtures, it did not change the baricity from hyperbaric to isobaric. However, the addition of highly hypobaric fentanyl 0.99360 g/mL (SD ± 0.00004) changes all isobaric LA solutions baricity to hypobaric. The comparison of measured and theoretical densities revealed significant differences (*p* > 0.05). However, the absolute reduction reached 0.0006 g/mL in only two LA-A compositions. We conclude that the addition of fentanyl to isobaric LA results in a hypobaric solution that may affect the distribution of the block. The inadequacy of LA-A in a clinical setting is unlikely to influence block characteristics.

## 1. Introduction

Spinal anesthesia is one of the most popular methods for regional anesthesia. It is the preferred method of anesthesia for cesarean delivery and is frequently used for other surgical interventions in the lower body [1,2]. The reasons for its popularity are the uncomplicated instrumentation, fast onset and reliability of the block, reduced need for airway instrumentation, lower risk of respiratory complications and reduced intraoperative blood loss [3,4]. This method also has a relatively short learning curve: approximately 45 attempts are necessary to achieve a 90% success rate [5].

However, intrathecal anesthesia also has disadvantages. Postdural puncture headaches are still an important clinical problem [6,7]. The inevitable degree of sympathetic block during spinal anesthesia may cause hemodynamic disturbances. It possesses the risk of minor (e.g., shivering) and severe adverse events (e.g., spinal abscess or hematoma) [8,9]. The most popular—single-shot spinal anesthesia—is non-titratable; thus, the block characteristics cannot be adjusted during surgery. Although the failure rate for intrathecal anesthesia is considered low, values between 1% and 17% have been reported [10,11].

Failed spinal anesthesia is defined as either a complete lack of block or deficiencies in its extent, quality or duration [10]. The reason may be multifactorial in origin and involve anatomical, technical and/or pharmacological issues [10]. One of them is the subarachnoid distribution of administered drugs. Typically, every spinal block is associated with intrathecal injection of local anesthetic (LA). Its spread depends on its density relative to the density of the cerebrospinal fluid (CSF), described by the term “baricity” [12]. Typically, the solutions of LA are either hyperbaric or isobaric, meaning their density is higher than or equal to that of CSF, respectively. Due to gravity, hyperbaric solutions are typically injected at the height of lumbar lordosis and distributed in the direction of the lowest points of thoracic or sacral kyphosis, which makes them more predictable than isobaric solutions in providing an adequate block [13]. Apart from LA, during spinal anesthesia and in the other methods of regional anesthesia, various adjuvants (A) may also be administered [14]. Their role is to prolong the duration of the block and improve its quality [15]. The most commonly used adjuvants are opioids, e.g., fentanyl and morphine. These drugs tend to activate receptors in the white and gray matter and bind to lipophilic structures in the epidural space [16]. Mixing LA with an adjuvant may influence the final density of such a solution (LA-A). Studies on this topic reveal a possible impact on block distribution [17]. However, the popularity of “low dose” and “fast track” spinal anesthesia justifies the re-evaluation of this topic. It is also important to verify the degree of the intrathecal solution density changes by using LA-A mixtures created with the use of “at the bedside” methods rather than highly accurate laboratory techniques. This would inform the clinical significance of the density change in spinal anesthesia. Verifying the accuracy of preparing LA-A solutions in the clinical setting can improve our knowledge on the topic of failed intrathecal blocks. The aim of this study was to verify the extent of baricity change after the addition of opioid adjuvants to various doses of iso and hyperbaric local anesthetics. The comparison of measured and theoretical densities of the obtained LA-A solutions was performed to examine the impact of discrepancies during LA-A solutions preparation.

## 2. Results

**Figure 1:**
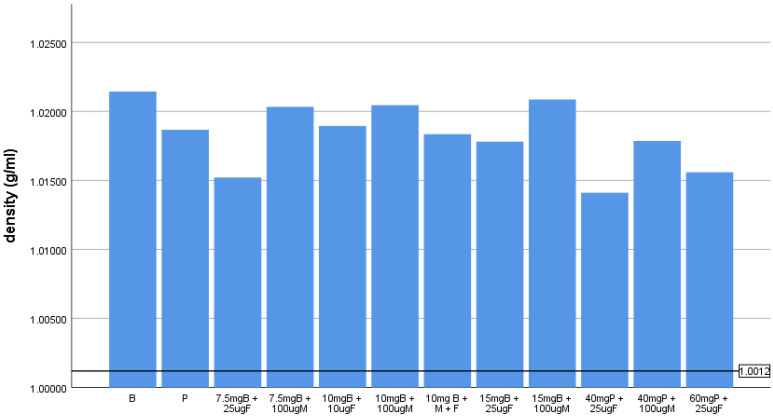
The density of the hyperbaric solutions relative to the upper limit of isobaricity. The black horizontal line represents the upper limit of isobaricity. B-bupivacaine, P-prilocaine, F-fentanyl, M-morphine.

The mean density and standard deviation of pure solutions of hyperbaric LA and LA mixtures with opioid adjuvants and combinations thereof with solutions are presented in Table 1 and shown graphically in Figure 1.

**Figure 2:**
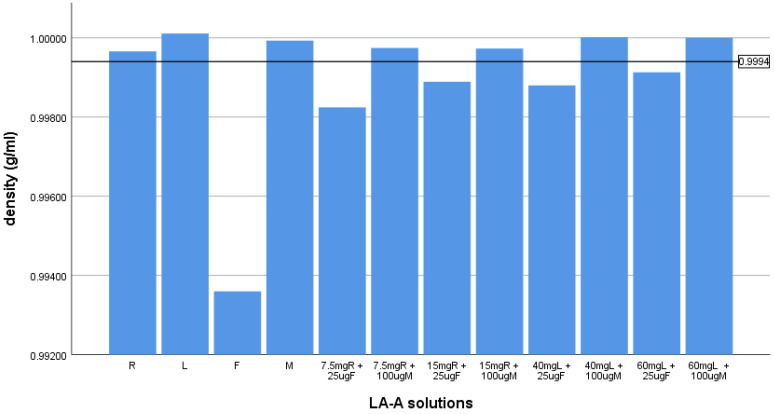
The density of the isobaric solutions relative to the lower limit of isobaricity. The horizontal dotted line represents the lower limit of isobaricity. R-ropivacaine, L-lidocaine, F-fentanyl, M-morphine.

In none of the LA-A combinations was the reduction in the density sufficient to change the baricity from hyperbaric to isobaric. However, in all cases, the density change exceeded 0.0006 g/mL. The results for isobaric solutions are presented in Table 2 and graphically in Figure 2.

The density changes of all mixtures of isobaric LA with fentanyl exceeded the presumed threshold of clinical significance. The use of fentanyl as an adjuvant caused all isobaric LA-A solutions to change the baricity of LA-A from isobaric to hypobaric.

Statistical analysis of each LA-A composition’s mean measured density and its theoretical density calculated with Hare’s formula revealed significant differences (*p* > 0.05) between those values in numerous cases. However, only two LA-A compositions, 7.5 mg hyperbaric bupivacaine with 25 µg of fentanyl and 60 mg hyperbaric prilocaine with 25 µg of fentanyl, exceeded the 0.0006 g/dL threshold.

## 3. Discussion

Our study confirms the already published observations that adding opioids as adjuvants to the local anesthetic solution in clinically relevant doses may affect the final density of such compositions [1,7,18]. This reduction in density may exceed the threshold of clinical significance at 0.006 g/mL. The importance of the obtained results is emphasized by the fact that we measured the density of solutions prepared in an exact way for intrathecal anesthesia in the operating room—each measurement was taken from a separate syringe. The published studies on this subject typically measure LA-A solution density mixed with the use of precise laboratory methods or by mixing compounds at an appropriate rate and multiplying by a set volume [17,18]. These methods differ and may be substantially more accurate than how LA-A solutions are prepared at the bedside.

There is a substantial difference between hyperbaric and isobaric LA solutions and their relation with the CSF density range. Hyperbaric bupivacaine and prilocaine solution densities are higher than the upper limit of the 99% confidence interval of CSF, and the difference reaches one decimal place. Isobaric lidocaine and ropivacaine mean densities are, on the contrary, lower than the mean value for CSF, with the latter being only 0.00026 g/dL from the lover limit of the CSF 99% confidence interval. CSF density may differ in certain groups of patients, being the lowest in pregnant and postpartum women and highest in men [19,20]. This suggests that ropivacaine solution at 37 °C may be clinically hypobaric among men.

The opioid adjuvant measurements revealed that pure fentanyl solution is highly hypobaric. Comparative values can be observed in other studies where the density of intrathecal opioids was examined [17,18]. This feature of fentanyl influenced the density and baricity of LA-A mixtures measured in the study. Combinations of opioids with various doses of hyperbaric LA resulted in a reduced density of the final mixture above 0.0006 g/mL; however, in all cases, solutions remained hyperbaric. In the case of combinations of isobaric LA with opioids, only fentanyl caused a clinically significant difference in the final density of the LA-A mixture. This observation can be explained by the fact that intrathecal morphine has a comparable density to lidocaine and ropivacaine. What is of particular importance is that all solutions of isobaric LA with fentanyl had densities that were measured to be below the lower 99% confidence interval of CSF density, rendering these solutions hypobaric. The 0.0006 g/mL density change as a threshold value for density change affecting LA-A dispersion is derived from experimental studies on spinal canal models, but data on the clinical significance of this threshold are sparse [21]. Published studies such as Paterson et al. do not reveal the clinically significant influence of density reduction on block characteristics [22]. However, if adjuvant administration causes a substantial change in LA-A’s relative baricity, it will result in a clinically different block distribution [23]. Although hypobaric solutions are used in anorectal surgery or unilateral blocks to potentially reduce the hemodynamic effects of a sympathetic block, their administration in the common sitting position used in our institution may result in higher spread and potentially more prominent features of a sympathetic block [24,25,26].

The second part of our analysis compared the expected density, as calculated with Hare’s formula, with measured values for various LA-As. Although all densitometrically obtained results were significantly different from estimations, these differences did not reach the clinical significance level apart from 7.5 mg Bupi + 25 µg Fentanyl and 60 mg Prilo + 25 µg fentanyl solutions. In our opinion, concerning the utility of the 0.0006 g/mL density change threshold, bedside preparation of LA-A is precise, and potential discrepancies are unlikely to be the cause of the inadequate block.

Our study has certain limitations. We could not include isobaric bupivacaine nor hyperbaric ropivacaine as they are not registered in our country (hyperbaric ropivacaine); thus, they are unavailable or not registered for spinal anesthesia (isobaric bupivacaine). We measured all densities at a temperature of 37 °C. The LA-A solutions are typically prepared and injected at a temperature of approximately 20 °C, and the injection temperature may affect the block distribution. As the equilibration of the injected solution to body temperature takes 2 min, we believe that values obtained at 37 °C are the most clinically important for final block distribution. Our study measured mixed LA and A compositions. Therefore, this study cannot provide information about the effects of a common technique in intrathecal anesthesia, which involves administering all compounds consecutively without mixing them before injection. Such a method may also result in different spinal anesthesia distributions [27]. Moreover, it is worth stressing that LA-A solution density is only one of the numerous factors affecting the final spread of the spinal block. Our study included only opioid adjuvants, however, in current practice other non-opioid additives are also being used through intrathecal route, i.e., alpha 2 agonists [28]. The reason was that those drugs are not registered for spinal anesthesia in our country and the authors decided to concentrate only on LA-A compositions used in everyday practice. It is worth it to note that some of the intrathecal additives have comparable effects when administered intravenously. This may help omit the effect of baricity change. However, current literature supports intrathecal route regarding opioid adjuvants [29,30].

In conclusion, the addition of an opioid adjuvant to a local anesthetic solution affects the final density of the intrathecal solution. Combinations of isobaric LA with fentanyl may result in the creation of a hypobaric solution that may affect the distribution of the block. The inadequacy of LA-A preparation methods used in a clinical setting is unlikely to influence spinal block characteristics.

## 4. Materials and Methods

The study took place between 18 February 2021 and 22 February 2021. The hyperbaric solutions of 0.5% bupivacaine (Marcaine Spinal Heavy) and 2% prilocaine (Prilotekal) and isobaric solutions of 0.5% ropivacaine (0.5% Ropimiol) and 2% lidocaine (Lignocainum Hydrochloricum WZF 2%) were mixed with solutions of opioid adjuvants: 0.05 mg/cm^3^ fentanyl (Fentanyl WZF) and 0.1% morphine (Morphini Sulfas WZF 0.1% Spinal). Local anesthetic and opioid adjuvant doses examined in the study were the standard dosages used for spinal anesthesia procedures performed at the University Clinical Center in Gdansk—MUG University Hospital. The exact proportions of prepared intrathecal mixtures are presented in Table 3. A dose of 10 mg of hyperbaric bupivacaine with both opioid adjuvants together was chosen to cover all the compositions commonly used for spinal bocks during caesarian section. A composition of 60 mg of hyperbaric prilocaine and morphine was not included in the study as the LA-A solution is not used clinically in MUG hospital.

Two anesthesiologists mixed each of the local anesthetics with appropriate adjuvants. Ten 5 mL syringes were prepared for each of the selected LA-A compositions using only the equipment available in the operating room and at the bedside.

The densities of all intrathecal drug mixtures were measured using an Anton Paar DMA 1001 densimeter (Anton Paar GmbH, Gratz, Austria) at 37 °C. Ten measurements for every LA-A composition were performed, each from a separate syringe. The density of plain solutions of the LA and adjuvants was also examined.

Baricity and its change were determined by comparing the LA-A mixture density with the value of CSF, determined to be 1.0003 (SD 0.0003) g/L based on a published study [12].

A solution was considered isobaric when its measured density was within a range of ±SD from the mean CSF value. The normal distribution is within the 99% confidence limit. As a reduction in density of 0.0006 g/L may impact the distribution pattern of the intrathecal drug mixture, resulting in various levels of the block, such a difference was presumed to be clinically significant [21]. LA-A mixture relative density changes from hyperbaric to isobaric and isobaric to hypobaric consecutively were also verified.

In the second part of the study, in order to assess the possible discrepancies and its clinical significance in the preparation of LA-A solutions with the use of typical bedside equipment, we calculated the theoretical density of each local anesthetic-adjuvant mixture using the formula proposed by Hare et al.

Density mixture = (Density local anesthetic-Density opioid) × fractional volume anesthetic + Density opioid [31].

Expected and measured values were compared by the one sample t-test. All calculations and statistics were made with the use of IBM SPSS statistics version 26 (Armonk, NY, USA: IBM Corp.).

## Figures and Tables

**Table 1:** Measured and calculated densities of hyperbaric LA-A solutions and difference from pure LA and calculated density. *—> 0.0006; #—*p* < 0.05.

	Measured Density g/mL (±SD)	Pure LA Density—LA-A Measured Density (g/mL)	Calculated Density (*p*-Value)	Measured Density—Calculated Density
**CSF**	1.0003 (0.0003)			
**Fentanyl**	0.99360 (0.00004)			
**Morphine**	0.99993 (0.00003)			
**Bupivacaine (Bupi)**	1.02143 (0.00002)			
**Prilocaine (Prilo)**	1.01867 (0.00009)			
**7.5 mg Bupi + 25 µg Fentanyl**	1.01513 (0.00053)	0.00630 *	1.01447 (0.003) #	0.00065
**7.5 mg Bupi + 100 µg Morphine**	1.02020 (0.00017)	0.00123 *	1.02009 (0.059)	0.00011
**10 mg Bupi + 10 µg Fentanyl**	1.01899 (0.00004)	0.00244 *	1.01890 (0.0001) #	0.00009
**10 mg Bupi + 100 µg Morphine**	1.02044 (0.00005)	0.00099 *	1.02041 (0.065)	0.00003
**10 mg Bupi + 100 µg Morphine + 10 µg Fentanyl**	1.01830 (0.00023)	0.00313 *	1.01808 (0.015) #	0.00022
**15 mg Bupi + 25 µg Fentanyl**	1.01770 (0.00047)	0.00373 *	1.01746 (0.144)	0.00024

**Table 2:** Measured and calculated densities of hypobaric LA-A solutions and difference from pure LA and calculated density. *—>0.0006; #—*p* < 0.05.

	Measured Density (g/mL) (±SD)	Pure LA Density—LA-A Measured Density (g/mL)	Calculated Density (*p*-Value)	Measured Density—Calculated Density
**CSF**	1.0003 (0.0003)			
**Lidocaine (Lido)**	1.00011 (0.00002)			
**Ropivacaine (Ropi)**	0.99966 (0.00009)			
**Fentanyl**	0.99360 (0.00004)			
**Morphine**	0.99993 (0.00003)			
**7.5 mg Ropi + 25 µg Fentanyl**	0.99824 (0.00011)	0.00142 *	0.99814 (0.016)	0.00010
**7.5 mg Ropi + 100 µg Morphine**	0.99975 (0.00005)	−0.00009	0.99968 (0.001) #	0.00008
**15 mg Ropi + 25 µg Fentanyl**	0.99890 (0.00010)	0.00076 *	0.99879 (0.007) #	0.00010
**15 mg Ropi + 100 µg Morphine**	0.99970 (0.00005)	−0.00004	0.99967 (0.11)	0.00003
**40 mg Lido + 25 µg Fentanyl**	0.99883 (0.00004)	0.00128 *	0.99881 (0.282)	0.00002
**40 mg Lido + 100 µg Morphine**	1.00002 (0.00004)	0.00009	1.00010 (0.0001) #	−0.00009
**60 mg Lido + 25 µg Fentanyl**	0.99915 (0.00006)	0.00096 *	0.99918 (0.187)	−0.00003
**60 mg Lido + 100 µg Morphine**	0.99999 (0.00004)	0.00012	1.00010 (0.00001) #	−0.00011

**Table 3:** Composition of LA-A solutions.

Local Anesthetic	Dose (Volume)	Adjuvant: Dose (Volume)					
		Isobaric Fentanyl 0.05 mg/mL		0.1% Morphine		Isobaric Fentanyl + 0.1% Morphine	
**0.5% hyperbaric bupivacaine**	7.5 mg (1.5 mL)	25 µg (0.5 mL)	2 mL	100 µg (0.1 mL)	1.6 mL	-	-
	10 mg (2 mL)	10 µg (0.2 mL)	2.2 mL	100 µg (0.1 mL)	2.1 mL	10 µg + 100 µg (0.2 mL) (0.1 mL)	2.3 mL
	15 mg (3 mL)	25 µg (0.5 mL)	3.5 mL	100 µg (0.1 mL)	3.1 mL	-	-
**0.5% isobaric ropivacaine**	7.5 mg (1.5 mL)	25 ug (0.5 mL)	2 mL	100 µg (0.1 mL)	1.6 mL	-	-
	15 mg (3 ml)	25 ug (0.5 mL)	3.5 mL	100 µg (0.1 mL)	3.1 mL	-	-
**2% hyperbaric prilocaine**	40 mg (2 mL)	25 ug (0.5 mL)	2.5 mL	100 µg (0.1 mL)	2.1 mL	-	-
	60 mg (3 mL)	25 ug (0.5 mL)	3.5 mL	-	-	-	-
**2% isobaric lidocaine**	40 mg (2 mL)	25 ug (0.5 mL)	2.5 mL	100 µg (0.1 mL)	2.1 mL	-	-
	60 mg (3 mL)	25 ug (0.5 mL)	3.5 mL	100 µg (0.1 mL)	3.1 mL	-	-

## Data Availability

The data presented in this study are available upon request from the corresponding author.
